# The Regulation of Nitric Oxide Synthase Isoform Expression in Mouse and Human Fallopian Tubes: Potential Insights for Ectopic Pregnancy

**DOI:** 10.3390/ijms16010049

**Published:** 2014-12-23

**Authors:** Junting Hu, Shulan Ma, Sien Zou, Xin Li, Peng Cui, Birgitta Weijdegård, Gencheng Wu, Ruijin Shao, Håkan Billig, Yi Feng

**Affiliations:** 1 Department of Integrative Medicine and Neurobiology, State Key Lab of Medical Neurobiology, Shanghai Medical College and Institute of Acupuncture Research (WHO Collaborating Center for Traditional Medicine), Institute of Brain Science, Fudan University, Shanghai 200032, China; E-Mails: 11211250005@fudan.edu.cn (J.H.); slma@shmu.edu.cn (S.M.); 13211010081@fudan.edu.cn (P.C.); gcwu@shmu.edu.cn (G.W.); 2 Department of Physiology and Endocrinology, Institute of Neuroscience and Physiology, the Sahlgrenska Academy at University of Gothenburg, 40530 Gothenburg, Sweden; E-Mails: Birgitta.Weijdegard@medfak.gu.se (B.W.); ruijin.shao@fysiologi.gu.se (R.S.); 3 Training Center of Medical Experiments, Fudan University, Shanghai 200032, China; 4 Department of Gynecology, Obstetrics and Gynecology Hospital of Fudan University, Shanghai 200011, China; E-Mails: zoushien@fudan.edu.cn (S.Z.); lxsure@fudan.edu.cn (X.L.)

**Keywords:** nitric oxide synthase, steroid hormones, Fallopian tube, ectopic pregnancy

## Abstract

Nitric oxide (NO) is highly unstable and has a half-life of seconds in buffer solutions. It is synthesized by NO-synthase (NOS), which has been found to exist in the following three isoforms: neuro nitric oxide synthase (nNOS), inducible nitric oxide synthase (iNOS), and endothelial nitric oxide synthase (eNOS). NOS activity is localized in the reproductive tracts of many species, although direct evidence for NOS isoforms in the Fallopian tubes of mice is still lacking. In the present study, we investigated the expression and regulation of NOS isoforms in the mouse and human Fallopian tubes during the estrous and menstrual cycles, respectively. We also measured isoform expression in humans with ectopic pregnancy and in mice treated with lipopolysaccharide (LPS). Our results confirmed the presence of different NOS isoforms in the mouse and human Fallopian tubes during different stages of the estrous and menstrual cycles and showed that iNOS expression increased in the Fallopian tubes of women with ectopic pregnancy and in LPS-treated mice. Elevated iNOS activity might influence ovulation, cilia beats, contractility, and embryo transportation in such a manner as to increase the risk of ectopic pregnancy. This study has provided morphological and molecular evidence that NOS isoforms are present and active in the human and mouse Fallopian tubes and suggests that iNOS might play an important role in both the reproductive cycle and infection-induced ectopic pregnancies.

## 1. Introduction

The Fallopian tube plays a central role in fertilization and early embryonic development by establishing the environment that facilitates the transport and maturation of gametes and embryos [1]. Gamete and embryo transport in the mammalian Fallopian tube results from a complex and still poorly understood interaction between smooth muscle contractions, cilia activity, and secretory functions [2]. The tubal wall is made mostly of smooth muscle cells that exhibit both phasic and tonic contractile activity, but little is known about how smooth muscle contractions are integrated into the process of tubal embryo transport [3]. In addition to smooth muscle, other factors such as adrenergic nerves, ovarian steroids, oxytocin, and prostaglandins are also involved in embryo transport [1].

The realization that nitric oxide (NO) acts as an endogenous signaling molecule under normal physiological conditions has had significant effects on scientific and medical research over the past 30 years [3]. NO is highly unstable and has a half-life of only a few seconds in buffer solutions [3], but it has been recognized as an important molecule that regulates the biology and physiology of the reproductive system and it has been shown to influence ovulation, cilia beats, contractility, relaxation, and the transport of gametes and embryos [1,2,4,5]. It is synthesized during the conversion of l-arginine to citrulline by the action of NO synthase (NOS), which exists as neuro nitric oxide synthase (nNOS), inducible nitric oxide synthase (iNOS), and endothelial nitric oxide synthase (eNOS) isoforms [6,7]. The genetic sequences for each isoform reside on three different chromosomes. nNOS and eNOS are constitutively expressed in the cytosol and facilitate the generation of NO for short time periods. The NO produced by these enzymes acts as a transduction mechanism that forms the basis of several different physiological responses. The iNOS isoform, on the other hand, can be induced after the activation of macrophages and can be induced in endothelial cells and a number of other cells by cytokines such as interleukin (IL)-1, IL-2, and IL-12; tumor necrosis factor alpha; and the endotoxin lipopolysaccharide (LPS). Once expressed, iNOS produces abundant NO in a calcium-independent manner for an extended period of time [8,9].

Damage and/or dysfunction of the Fallopian tube can trigger ectopic pregnancy, which is the major cause of maternal morbidity and mortality in early pregnancy [10]. Over 95.5% of extra-uterine pregnancies are located in the Fallopian tube [11] where the embryo is often aborted, ruptured, or stops growing [12]. Multiple factors contribute to the risk of ectopic pregnancy, including pelvic inflammatory disease, infection, increased age at conception, smoking, and *in vitro* fertilization [13,14,15,16,17].

In the present study, we investigated the expression of NOS in the Fallopian tubes of mice and humans at different stages of the reproductive cycle and studied whether NOS isoforms were hormonally regulated in the human and mouse Fallopian tubes. We also measured the expression of different NOS isoforms in the implantation and non-implantation sites of Fallopian tubes collected from women with ectopic pregnancies. Finally, we explored whether iNOS expression was up-regulated in the Fallopian tubes of mice after exposure to LPS, which has been shown to be one possible risk for ectopic pregnancy [18].

## 2. Results

### 2.1. Nitric Oxide Synthase (NOS) Isoform Expression during the Estrous Cycle in Mouse Fallopian Tubes

**Figure 1:**
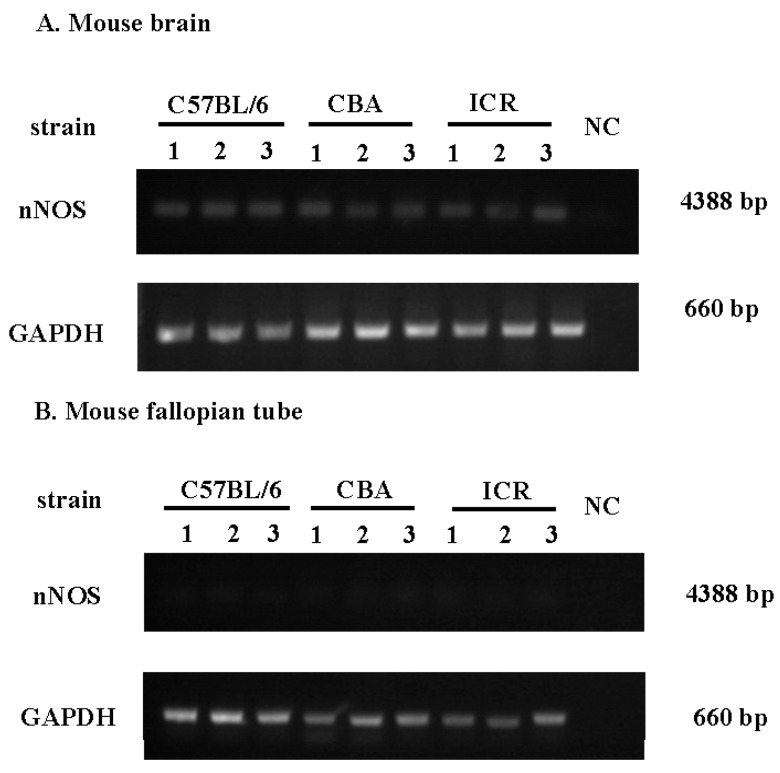
RT-PCR of neuro nitric oxide synthase (nNOS) in mouse brains and Fallopian tubes. (**A**) The gel electrophoresis of the RT-PCR products for nNOS and GAPDH in the brains of C57BL/6 mice, CBA mice and ICR mice; and (**B**) The gel electrophoresis of the RT-PCR products for nNOS and GAPDH in the Fallopian tubes of C57BL/6 mice, CBA mice, and ICR mice. Samples from three mice are shown in each lane 1–3. NC: negative control.

**Figure 2:**
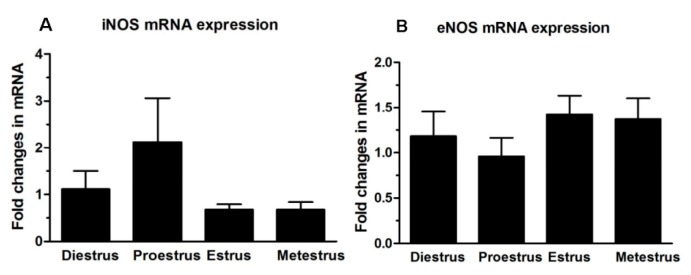
Quantitative RT-PCR analysis of iNOS (**A**) and eNOS (**B**) mRNA levels in adult mouse Fallopian tubes at different stages of the estrous cycle (*n* = 10/group). mRNA levels of each gene were relative to 18S rRNA levels in the same samples. Significance was tested by one-way ANOVA with Dunnett’s test, and no significant differences were found between any groups. Values are expressed as mean ± SEM.

In this study, nNOS was not detected in the Fallopian tubes of C57/BL6 mice by qRT-PCR. To further confirm this, nine brains and Fallopian tubes from three strains of mice (C57/BL6, CBA, and ICR) were used to study the expression of nNOS by RT-PCR. The results showed that nNOS was expressed only in the mouse brain, and not in the mouse Fallopian tube (Figure 1). Both iNOS and eNOS mRNAs were abundant in the mouse Fallopian tube and were expressed in all stages of the estrous cycle, but there was no statistical difference between iNOS and eNOS mRNA levels (Figure 2).

**Figure 3:**
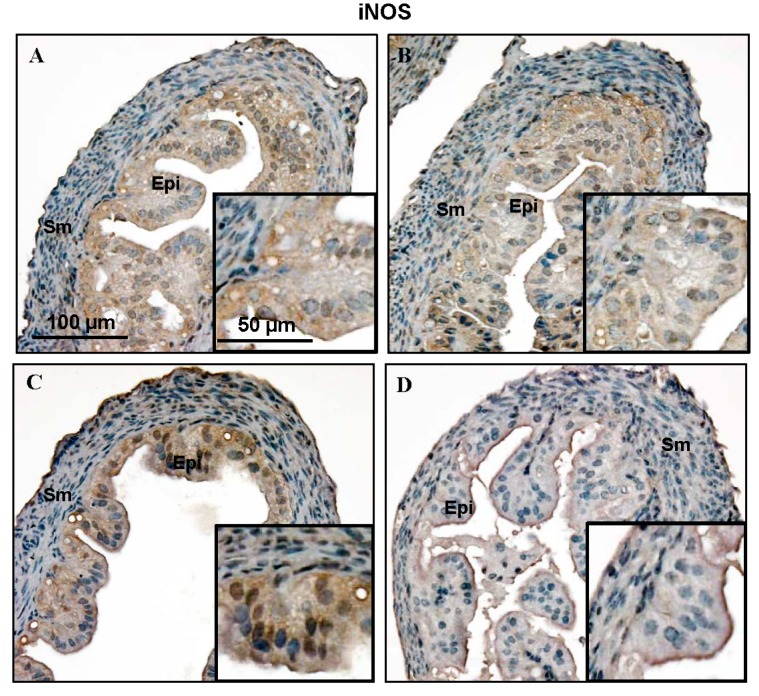
Immunochemical detection of the cellular distribution of iNOS expression in the mouse Fallopian tube at different stages of the estrous cycle (**A**–**D**) with diaminobenzidine (DAB). All slides were counterstained with hematoxylin. Enlarged views of one part are shown in the lower right corner in each picture. Epi: epithelial cells; Sm: smooth muscle cells. (**A**) diestrus; (**B**) proestrus; (**C**) estrus; (**D**) metestrus. Scale bar = 100 µm in the main picture and 50 µm in the enlarged images.

**Figure 4:**
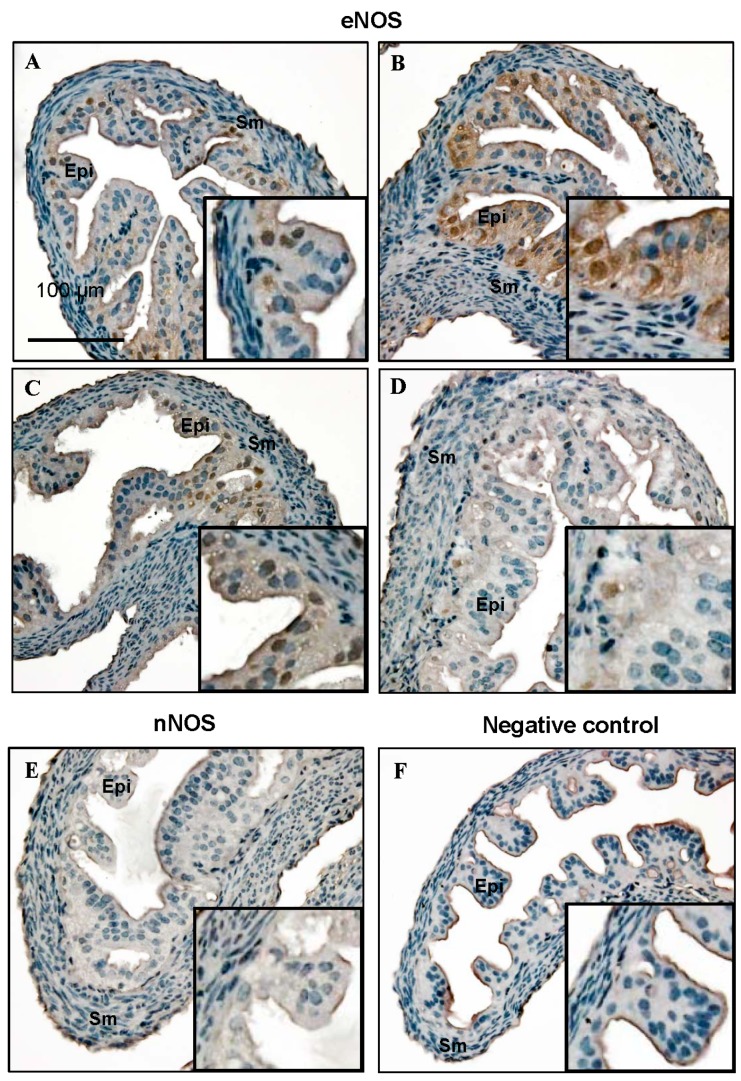
The eNOS expression in the mouse Fallopian tube by immunochemical methods and diaminobenzidine (DAB) staining. All slides were counterstained with hematoxylin. Enlarged views of one part are shown in the lower right corners in each picture. Epi, epithelial cells; Sm, smooth muscle cells; (**A**) Diestrus; (**B**) Proestrus; (**C**) Estrus; (**D**) Metestrus; (**E**) nNOS expression in the proestrus stage; and (**F**) Negative control using goat serum instead of the primary antibody showing the absence of staining. Scale bar = 100 µm.

Immunochemistry showed that both iNOS (Figure 3A–D) and eNOS (Figure 4A–D) were expressed in the mouse Fallopian tube at all four stages of the estrous cycle, and there were clear differences in immunoreactivity density and quantity at the different stages of the estrous cycle. The intensity of iNOS was highest in the estrus stage (Figure 3C) while eNOS was most abundant in the proestrus stage (Figure 4B). Both of them showed the weakest signal in the metestrus stage (Figure 3D and Figure 4D). iNOS and eNOS protein were mostly located in the epithelium of mouse Fallopian tubes and were found in both the cytoplasm and in the nucleus. However, they were not detectable in every epithelial cell. iNOS was expressed in about half of the epithelial cells, and eNOS was only detectable in about 1/3 of the epithelial cells. Similar to the mRNA results, there were no nNOS-positive cells in any of the Fallopian tubes. As a negative control, normal goat serum replaced the primary antibody (Figure 4F).

### 2.2. Hormones Directly Regulate NOS Isoform Expression in the Mouse Fallopian Tube

**Figure 5:**
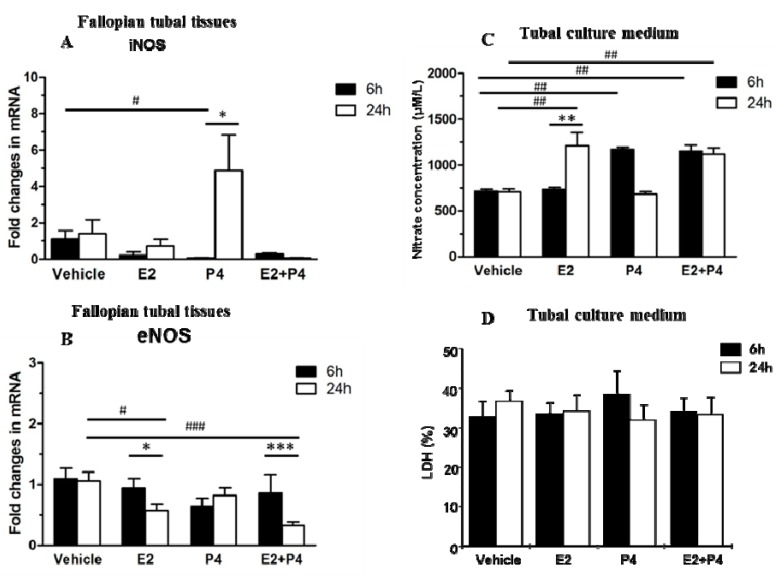
Time-dependent regulation of iNOS and eNOS expression in mouse Fallopian tubes following steroid hormone treatment. (**A**) The iNOS mRNA level in mouse Fallopian tubes *in vitro*. The expression of iNOS mRNA was significantly up-regulated from 6 to 24 h following P4 treatment but not following E2 or E2 plus P4 together (*p* < 0.05); (**B**) The eNOS mRNA level in mouse Fallopian tubes *in vitro*. The expression of eNOS mRNA was down-regulated from 6 to 24 h following E2 and E2 plus P4 treatment together but not after P4 treatment; (**C**) The nitric oxide concentration in tubal culture medium *in vitro*. The nitric oxide concentration was significantly lower after 6 h in tissue cultured with P4 or E2 plus P4. The nitrate concentration was lower after 24 h in culture medium containing E2 and E2 plus P4; and (**D**) The LDH level in the tubal culture medium as measured by the optical density on a microplate fluorometer at a wavelength of 490 nm. There was no significant difference between the four groups. Significance was tested by one-way ANOVA or two-way ANOVA. Values are expressed as mean ± SEM. * *p* < 0.05, ** *p* < 0.01, *** *p* < 0.001 compared with 6 h incubation of same steroid hormone treatment; ^#^ *p* < 0.05, ^##^ *p* < 0.01, ^###^ *p* < 0.001 compared with vehicle at the same incubation time.

To determine whether ovarian steroid hormones regulate NOS expression in mouse Fallopian tubes, qRT-PCR was performed in mouse Fallopian tubes treated with E2 or P4 *in vitro.* The expression of iNOS mRNA was significantly up-regulated from 24 h following P4 treatment but not following E2 or a combination of E2 and P4 (*p* < 0.05), but compared with vehicle, iNOS mRNA level was decreased with 6 h P4 treatment (*p* < 0.05) (Figure 5A). In contrast, the expression of eNOS mRNA was down-regulated from 6 to 24 h following treatment with E2 (*p* < 0.05) and the combination of E2 and P4 (*p* < 0.001), but not P4. In addition, eNOS mRNA level was decreased with 24 h E2 (*p* < 0.05) and E2 plus P4 (*p* < 0.001) treatment compared with vehicle (Figure 5B).

### 2.3. NO Levels and Lactate Dehydrogenase (LDH) Release Levels in Mouse Fallopian Tube Cultures

NO was synthesized by NOS and was determined by measuring the nitrate concentration in the *in vitro* Fallopian tube culture medium. Compared with the vehicle, NO concentration was significantly higher after 6 h with P4 or E2 plus P4 (*p* < 0.01) and 24 h with E2 or E2 plus P4 (*p* < 0.01) in culture medium. Moreover, the nitrate concentration was increased significantly in culture medium containing E2 at 24 h compared to 6 h (*p* < 0.01) (Figure 5C). In contrast, there was no significant difference in lactate dehydrogenase (LDH) release between the four treatment groups (Figure 5D).

### 2.4. NOS Isoform Expression in Human Fallopian Tubes during the Menstrual Cycle

**Figure 6:**
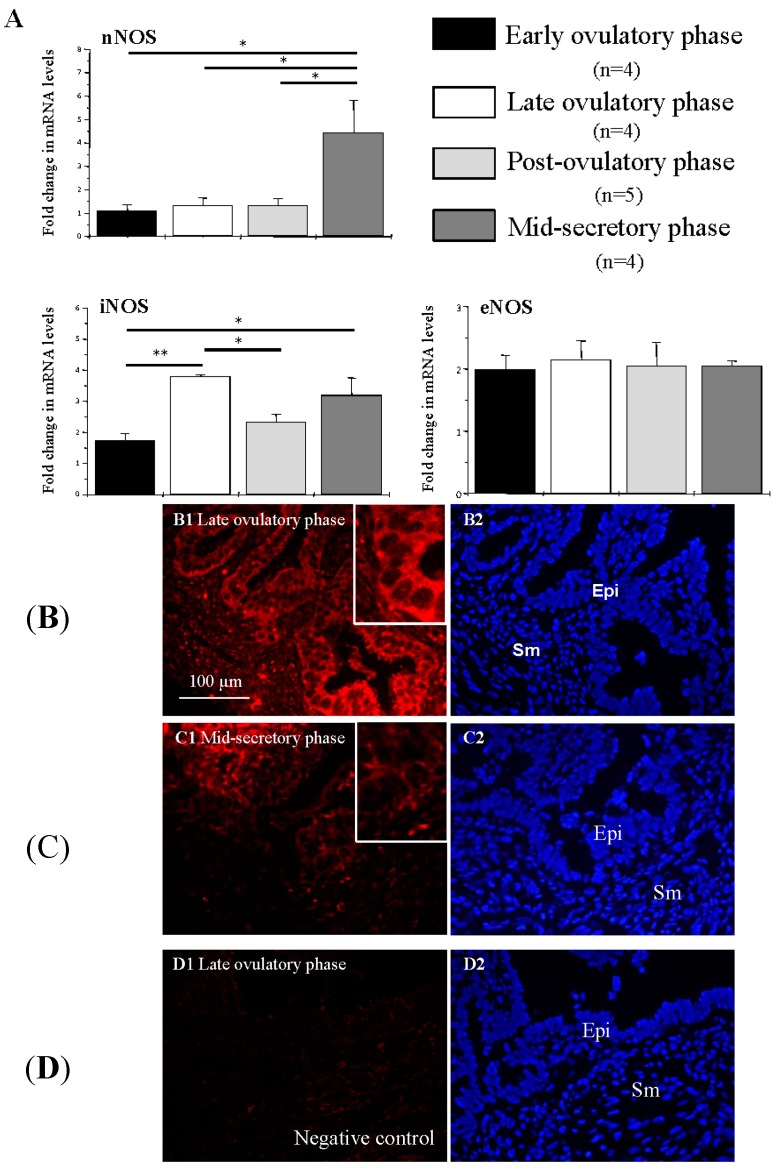
The mRNA and protein levels of the NOS isoforms in human Fallopian tubes. (**A**) Fold changes in nNOS, iNOS, and eNOS mRNA levels at different stages of the menstrual cycle. * *p* < 0.05, ** *p* < 0.01; (**B**,**C**) iNOS expression in late ovulatory phase and middle secretory phase. Slides were subsequently counterstained with DAPI to visualize the cell nuclei. Sections were immunolabeled for iNOS (red) and DAPI (blue). iNOS was found both in the epithelial cells and muscle cells of the human Fallopian tube and was located in the cytoplasm of the epithelial cells. The amount and density of iNOS expression in the late ovulatory phase was higher than in the mid-secretary phase; and (**D**) Negative control using goat serum instead of primary antibody resulted in no immunostaining. Epi, epithelial cells; Sm, smooth muscle cells. Scale bar = 100 µm.

mRNAs of all three NOS isoforms were expressed in the human Fallopian tube (Figure 6A). The nNOS mRNA level was increased in the mid-secretory stage compared with the other stages (*p* < 0.05). The iNOS mRNA level was increased in the late ovulatory phase compared with the early ovulatory phase (*p* < 0.01) and post-ovulatory phase (*p* < 0.05), and the iNOS mRNA level in the mid-secretory phase was higher than in the early ovulatory phase (*p* < 0.05). However, there were no significant changes in eNOS mRNA levels at the different stages. Immunochemical analysis showed that iNOS was found in both the epithelial cells and muscle cells of the human Fallopian tube. It was located only in the cytoplasm of the epithelial cells but in both the cytoplasm and nucleus in muscle cells. Both the quantity and the density of iNOS expression in the late ovulatory phase was much higher than in the mid-secretory phase (Figure 6B,C).

### 2.5. NOS Isoform Expression during Human Ectopic Pregnancy

**Figure 7:**
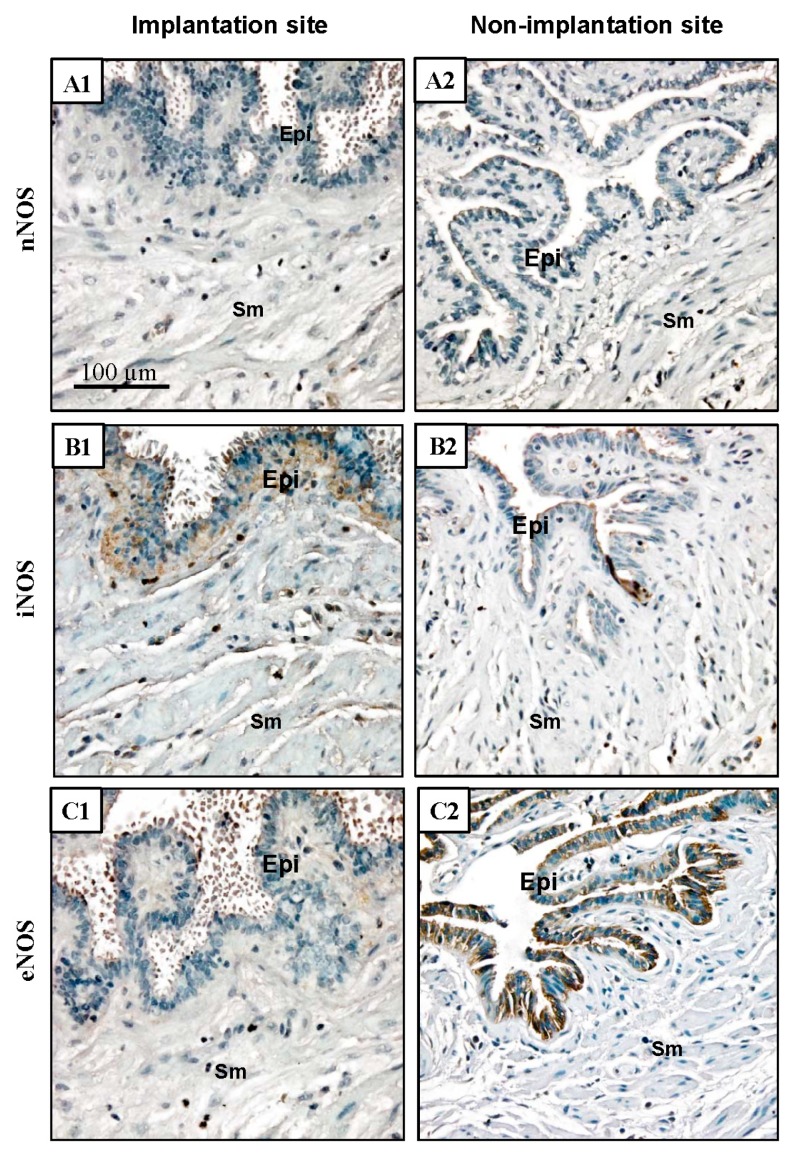
NOS expression in the human Fallopian tube during ectopic pregnancy. (**A1**,**A2**) nNOS expression in the implantation site and non-implantation site; (**B1**,**B2**) iNOS expression in the implantation site and non-implantation site; (**C1**,**C2**) eNOS expression in the implantation site and non-implantation site. Epi, epithelial cells; Sm, smooth muscle cells. Scale bar = 100 µm.

Both the quantity and density of iNOS was higher in the implantation site than the non-implantation site. However, eNOS levels were lower in the implantation site and nNOS showed no change. Both iNOS and eNOS were expressed in the epithelial cells (Figure 7).

### 2.6. Increased iNOS Expression in Mice Treated with Lipopolysaccharide (LPS)

**Figure 8:**
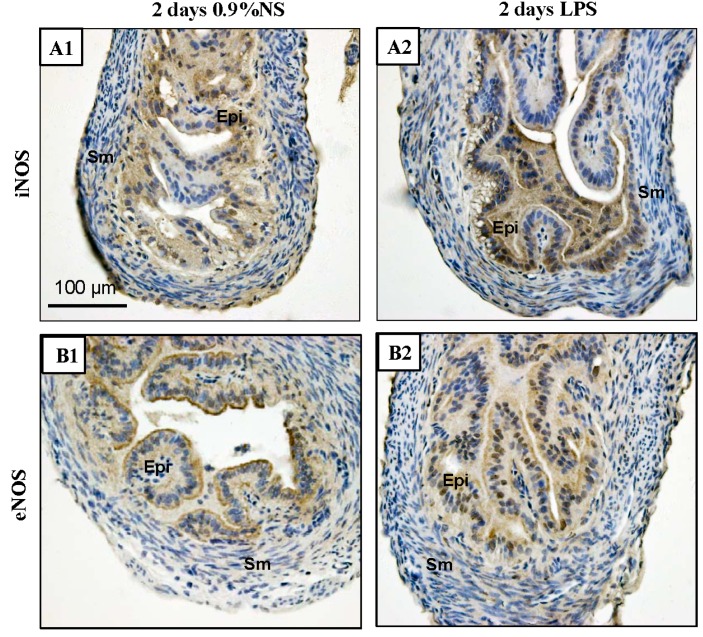
Immunochemical detection of iNOS and eNOS expression in the mouse Fallopian tubes after injection of LPS for two days. (**A1**,**A2**) iNOS expression in Fallopian tubes of mice treated with 0.9% NS compared with LPS (1 mg/kg body weight). iNOS expression was stronger in LPS-treated group and present in both the cytoplasm and nuclei of the epithelial cells; (**B1**,**B2**) eNOS expression in Fallopian tubes of mice treated with 0.9% NS compared with LPS. eNOS was also expressed in both the cytoplasm and nuclei of the epithelial cells, but there was no obvious change in eNOS expression in the Fallopian tubes of the LPS-treated mice compared to the 0.9% NS. Epi, epithelial cells; Sm, smooth muscle cells; NS, normal saline. Scale bar = 100 µm.

We reproduced an inflammatory model in mice by intraperitoneal injection of 1 μg/mg LPS and used immunohistochemistry to investigate iNOS and eNOS expression in the Fallopian tubes. The expression of iNOS was stronger in the Fallopian tubes of mice treated with LPS compared to controls. iNOS was present in both the cytoplasm and nuclei of the epithelial cells. eNOS was also expressed in both the cytoplasm and nuclei of the epithelial cells, but there was no obvious change in eNOS expression in the Fallopian tubes of the LPS-treated mice compared to the control group (Figure 8).

## 3. Discussion

The present study is the first to show that NOS isoforms are differentially expressed and regulated in mouse and human Fallopian tubes during the reproductive cycle. Steroid hormones directly regulated iNOS and eNOS in mice, and iNOS expression increased in the Fallopian tubes of women with ectopic pregnancies and in LPS-treated mice. These observations suggest that NOS variations exist in human and mouse Fallopian tubes and that iNOS in particular might play an important role in both the reproductive cycle and infection-induced ectopic pregnancies.

NOS isoform expression was shown to be cell-type specific in both mice and humans. iNOS and eNOS in mice were mainly located in the epithelial cells, and expression was both cytoplasmic and nuclear. nNOS was absent in the mouse Fallopian tube. However, all isoforms were expressed in epithelial cells of the human Fallopian tube but only iNOS was expressed in muscle cells. This suggests that NOS-induced NO production might be involved in the physiological function of the Fallopian tube, such as contractility. iNOS may be involved in the increases of ciliary activity and the transport of the oocyte to the ampulla in bovine fallopian tubes [19].

Although gene expression of NOS isoforms depends on various factors, hormonal regulation is the most influential in the Fallopian tube. Expression of NOS isoforms and nicotinamide adenine dinucleotide phosphate-diaphorase (a non-specific marker for all NOS isoforms) has been shown to vary during the reproductive cycle in human, rat, and bovine Fallopian tubes [6,19,20,21,22,23,24]. However, either only one isoform or no tissue-specific localization was described in the previous reports. Our results confirmed that NOS isoforms in the Fallopian tube are directly regulated by hormone fluctuations during the reproductive cycle in both mice and humans. In mice, the expression of iNOS protein was highest at the estrus stage, while eNOS was the most abundantly expressed at the proestrus stage. In our study, changes in NOS protein levels were not always accompanied by changes in the corresponding mRNAs in the Fallopian tubes *in vivo*. At this stage, it is not clear whether transcription and translation rates for individual NOS isoforms are different. On the other hand, although iNOS and eNOS mRNA levels did not show the statistical difference between the stages of estrous cycle, iNOS mRNA peaked at proestrus while its protein was increased at estrus. Meanwhile, eNOS mRNA was high at diestrus while its protein was increased at proestrus. The delay of protein peak may reflect the synthesis time from NOS mRNA to its protein. In humans, nNOS mRNA was highest at the mid-secretory stage and iNOS was highest at the late ovulatory phase. From these results, we can see different isoform of NOS might be regulated by different steroid hormones. Tubal NOS expression was easily regulated by steroid hormones such as E2 and P4, and clinical research has shown that increased levels of estrogen are related to circulating nitrate levels during follicular development in fertile women [25,26]. For patients with *in vitro* fertilization (IVF) treatment, the circulating estrogen level correlates with circulating and follicular levels of nitrite and nitrate during follicular stimulation [25]. In bovines, E2 selectively up-regulates iNOS mRNA and protein expression in the Fallopian tube at the periovulatory stage [19]. In contrast to estrogen, the NOS isoform expression during pregnancy did not prove the P4-mediated induction of nNOS and eNOS, but tubal iNOS mRNA and protein levels were increased by P4 in pregnant rodents *in vivo* [9]. Moreover, P4 significantly stimulated iNOS and eNOS mRNA levels in bovine oviduct epithelial cells *in vitro* [23]. Our results, together with those from other laboratories, suggest that the existence of different steroid hormonal regulation of NOS expression in the Fallopian tube is possibly due to the use of different animals and experimental setting (normal estrous cycle *vs.* pregnancy, and *in vivo vs. in vitro*).

To better understand which steroid hormones directly regulate NOS isoforms in mouse Fallopian tubes, we used the Fallopian tubes from 21-day-old mice as a non-steroid stimulated *in vitro* model. After exposure to P4 for 24 h, iNOS mRNA expression was up-regulated in the Fallopian tube, and after exposure to E2 eNOS mRNA expression was rapidly down-regulated. These results suggest that estrogen might down-regulate transcription of eNOS from mRNA to enzyme, but P4 might selectively up-regulate iNOS mRNA transcription. The discrepancy between the NO production and NOS mRNA expressions under 24 h culture conditions for tubal tissues treated with different hormones is likely due to the measurement of indirect NO level in the culture medium and the different synthesis time of tubal NOS isoforms and reductase. NOS activity is highly controlled in mammals because its product NO is a potential regulator, and even acts as a hormone, in many biologic processes [4]. A large body of evidence suggests that NO functions to regulate tubal contractility and relaxation under physiological conditions [4,27]. Two NO donors (nitroglycerin and spermine NONOate) and 8-bromo cGMP decrease the contractility of the human Fallopian tube *in vitro* [28], and *N*-nitro-l-arginine methyl ester (l-NAME), an inhibitor of NO synthesis, has the opposite effect in both human and bovine Fallopian tubes [3,25]. Moreover, local administration of spermine NONOate abolishes the effects of l-NAME and reduces the transport speed of ovulated oocyte-cumulus complexes in rat Fallopian tubes [2].

Gene knockout studies in mice have led to a better understanding of the function of each NOS isoform. Brain-specific nNOS knockout mice only retain about 0.3% of the nNOS activity in the brain, and both male and female mice are infertile. nNOS-deficient female mice exhibit neuroendocrine disorders and have smaller ovaries and fewer corpora lutea. However, transplantation of the ovaries from nNOS-knockout mice into wild-type mice leads to the return of normal ovulation under hormonal control. Therefore, nNOS in the central nervous system is crucial for ovulation [29]. iNOS-deficient female mice show reduced decidual cellular area and abnormally thick walls in the decidual arteries during mid-gestation, and this negatively affects embryo survival and pups size [30]. eNOS-deficient female mice have a reduced ovulatory capacity, a prolonged estrous cycle, impaired early embryonic viability, and decreased implantation during pregnancy [31,32]. In addition, nNOS/iNOS, nNOS/eNOS, and iNOS/eNOS double homozygous mutant female mice also have irregular estrous cycles and reduced fertility [33]. However, the effects of NOS isoforms, individually or in combination, on Fallopian tube function are poorly understood.

Isoform-specific NOS-deficient mouse models have also been shown to have altered immune responses [34]. The most interesting are iNOS-deficient mice that show increased susceptibility to bacterial and viral pathogens and increased sensitivity to cytokines and endotoxins [20,35,36]. In fact, inflammation, especially that caused by Chlamydia infection, is the major cause of human ectopic pregnancy [13,37]. The Fallopian tubes are the primary targets for Chlamydia infection [16] and might serve as the first line of defense against infection by regulating both innate and adaptive immune responses [38]. Indeed, it has been suggested that initial alterations in tubal cell mobility—which result in abnormal ciliary activity, epithelial secretion, and contractility—might be responsible for ectopic pregnancy. Epithelial cells and macrophages are the targets of *Chlamydia trachomatis* infection in the Fallopian tube [39], and these are the major cellular sites for the disease. Interestingly, we found that iNOS was specifically expressed in epithelial cells where it regulates NO production through autocrine or paracrine effects. Notably, in mice with *C. muridarum* infection, the transport of ovulated oocyte-cumulus complexes is inhibited by loss of spontaneous contractile activity and up-regulation of iNOS protein expression in Fallopian tubes *in vivo* [40].

Although the mechanisms linking *C. trachomatis* infection, iNOS, and tubal tissue/cell destruction are not completely understood, there is evidence that chronic and intense inflammation contributes to tissue remodeling and scarring in the Fallopian tube [41,42], which increases the risk for tubal ectopic pregnancy. In addition, Marconi *et al.* found increased cervicovaginal IL-1β, IL-6, and IL-8 levels in women with *C. trachomatis* infection [43]. In the present study, we have found an increase in the expression of the iNOS isoform in the human Fallopian tube with ectopic pregnancy compared with controls. We obtained the same result with mice treated with LPS, and this suggests that iNOS might play an important role in inflammation-induced ectopic pregnancy. Cytokines produced in the Fallopian tube in response to Chlamydia infection might regulate iNOS and subsequently generate NO. In fact, NO is considered to be part of the innate immune response because it is a bactericidal agent that is lethal to intracellular pathogens such as *C. trachomatis*. NO production is also a feature of innate immune cells such as macrophages and dendritic cells [44].

NO production in response to *C. trachomatis* infection in the Fallopian tube can be both protective and pathogenic. NO can participate in microbe-triggered immune responses within the Fallopian tube; however, excess levels of NO might lead to tubal damage and ectopic pregnancy [10]. Although the cause of tubal ectopic pregnancy seems to be multifactorial, NO and iNOS are likely to contribute to the etiology of ectopic pregnancy because tubal iNOS mRNA and protein expression is increased in the ampullary region, a site of fertilization, in women with tubal ectopic pregnancy [6]. Recently, eNOS expression was reported to be increased in tubal ectopic pregnancy compared with healthy women [45], but we did not observe a similar result.

The present study has provided morphological and molecular evidence that NOS in the human and mouse Fallopian tubes might participate in tubal function and suggests that iNOS might be involved in inflammation-induced ectopic pregnancies and, therefore, might be a possible target for non-surgical interventions.

## 4. Materials and Methods

### 4.1. Experimental Animals

All animal experiments in this study were approved by the ethics committee of Fudan University. Twenty-one-day-old and two-month-old female C57BL/6, CBA, and ICR mice were obtained from the Shanghai Laboratory Animal Center (SLAC, Shanghai, China). Animals were kept in groups with free access to food and water and a controlled temperature of 22 ± 2 °C with a 12 h light/dark cycle. Adult mice were confirmed by examination of vaginal smears under a light microscope for two sequential estrous cycles (about 8–9 days).

Mice were sacrificed and their Fallopian tubes were immediately removed and were either fixed in 4% paraformaldehyde overnight at 4 °C for paraffin embedding or flash frozen in liquid nitrogen.

For LPS treatment, adult mice were divided into two groups of six animals each. The experimental group was administered LPS (1 mg/kg body weight) in 100 μL sterile saline by intraperitoneal injection on two consecutive days. The control group received 100 μL saline only. LPS-induced infection models were studied by evaluating the status of the Fallopian tubes after two days of injection. The animals were killed by cervical dislocation after two days, and their Fallopian tubes were collected immediately. Tissues were dissected out, freed from fat, and fixed in formalin for 24 h.

### 4.2. Human Tissues

The human study was approved by the Human Research Ethics Committee of the Gynecology and Obstetrics Hospital of Fudan University (20100324, 24 March 2010, The signaling pathways in the regulation of tubal function in health and disease.) and by Animal Research Ethics of Shanghai Medical College of Fudan University (20120302-118, 2 March 2012, How Fallopian tubal dysfunction contributes to the initiation and development of human ectopic pregnancy: A translation approach for experimental proof to clinical practice.) and was conducted at the Sahlgrenska Academy at the University of Gothenburg in accordance with the Declaration of Helsinki for medical research involving human subjects. All patients provided informed consent to participate in the study.

The normal Fallopian tube samples were obtained from 17 women admitted for sterilization or hysterectomy. The inclusion criteria were regular menstrual cycles (25–29 days), age 28–37 years, proven fertility (at least one child, mean 2.9 children), no chronic systemic disease, and no hormonal therapy for at least 3 months prior to surgery. All patients were monitored by serial transvaginal ultrasound (Aloka SSD-900/2000; Aloka, Tokyo, Japan) for at least one menstrual cycle (mean two cycles) before surgery to determine whether follicular development occurred. Women received a subcutaneous injection of recombinant human choriogonadotropin (rhCG) (250 µg Ovitrelle; Serono International, Geneva, Switzerland) to mimic the natural peak in luteinizing hormone when the dominant follicles were at least 14 mm and no more than 20 mm (mean ± SEM, 17.1 ± 0.3 mm) [46]. The ovulatory stage was determined by the following: the early phase was defined as 12–18 h after rhCG injection (*n* = 4), the late phase as 18–24 h after rhCG (*n* = 4), and the postovulatory phase as 24–45 h after rhCG (*n* = 5). The mid secretory phase (*n* = 4) was determined from the last menstrual period and by endometrial histology. Serum was obtained immediately before surgery to confirm the ovulatory and mid secretory phases.

To further our understanding of how NOS affects human ectopic pregnancy, we dissected the implantation site of human Fallopian tube and the non-implantation site (1 cm away from the ipsilateral implantation site of the same patient) from five patients undergoing surgery to remove an ectopic pregnancy. These patients were from the Obstetrics and Gynecology Hospital of Fudan University.

All samples were washed with ice-cold RNase-free PBS and either snap-frozen in liquid nitrogen and stored at −80 °C for quantitative RT-PCR analysis or fixed in 4% formaldehyde for immunohistochemistry.

### 4.3. Tissue Culture and Hormonal Treatment

Twenty-one-day-old mice have low levels of ovarian steroid hormones and no hormone surges compared with mice after puberty or adult mice. Therefore, we used the Fallopian tubes of 21-day-old mice to observe the effect of exogenous 17β-estradiol (E2) and progesterone (P4) on NOS mRNA levels. Due to the very small size of the Fallopian tubes of the mouse pups, we pooled six Fallopian tubes (from three mice) to obtain sufficient amounts of total mRNA. Six pooled samples were treated with 17β-estradiol (E2), P4, a combination of E2 plus P4, or vehicle alone (sesame oil). The same concentrations of E2 (10 nM; Sigma–Aldrich, St. Louis, MO, USA) and P4 (100 nM; Sigma–Aldrich) were used according to a previous study [47]. After 6 or 24 h of incubation, the tissues were collected and immediately frozen in liquid nitrogen and stored at −80 °C for quantitative RT-PCR.

### 4.4. Immunochemistry and Immunofluorescence

Paraffin sections (4 μm) were used for immunochemical staining. After deparaffinization and re-hydration, the sections were treated with 10 mM sodium citrate buffer (pH 6.0; 10 min in a 700 W microwave) to retrieve the antigen. The sections were then incubated with 0.3% H_2_O_2_ for 15 min followed by 5% normal goat serum for 1 h at room temperature to reduce endogenous peroxidase and non-specific binding. The sections were incubated with primary antibodies (nNOS, 1:50 dilution, sc-5302, Santa Cruz Biotechnology, Dallas, TX, USA; iNOS, 1:100 dilution, sc-651, Santa Cruz Biotechnology; eNOS, 1:100 dilution, #9586, Cell Signaling Technology, Danvers, MA, USA) overnight at 4 °C. Sections were stained using the avidin-biotinylated-peroxidase complex detection system (ABC kit; Vector Laboratories, Burlingame, CA, USA) according to the manufacturer’s instructions, and the reactions were developed with 3,3-diaminobenzidine tetrahydrochloride (Sigma, St. Louis, MO, USA) [48]. Sections were examined on a Nikon E-1000 microscope (Nikon, Tokyo, Japan) under bright-field optics, and control slides incubated without the primary antibodies were always blank.

For immunofluorescence, sections were incubated with secondary antibody (Alexa Fluor 488 goat anti-rabbit IgG, 1:250 dilution; Alexa Fluor 594 goat anti-mouse IgG, 1:250 dilution, Invitrogen, Carlsbad, CA, USA) at room temperature for 1 h [48]. Slides were observed with a Nikon E-1000 microscope. Negative controls were treated the same way as above, but the primary antibodies were omitted.

### 4.5. RNA Extraction and Quantitative Real-Time PCR Analysis

Total cellular RNA from the Fallopian tubes was isolated with the RNeasy Micro Kit (74004, Qiagen, Hilden, Germany). The RNA concentration was estimated on a NanoDrop spectrophotometer (2000c, Thermo Fisher Scientific Inc., Waltham, MA, USA). Single-stranded cDNA was synthesized from each sample (10 μg) with the Quantscript Reverse Transcriptase Kit (KR103-04, Tiangen, Beijing, China). The resulting cDNA was amplified, and quantitative real-time RT-PCR (qRT-PCR) was performed on a Bio-Rad iQ5 using the Real Master Mix (FP202-02, Tiangen, Beijing, China) [11]. The primers were: 5'-CAA CAG CGT CTC CTC CTA TTC T-3' and 5'-TCA TCT CCC TCC CTC ATC TT-3' for nNOS; 5'-GCA AAC CCA AGG TCT ACG TTC A-3' and 5'-GAG CAC GCT GAG TAC CTC ATT G-3' for iNOS; 5'-GCA CAA GAG CTA CAA AAT CCG ATT-3' and 5'-GCC GCC AAG AGG ATA CCA-3' for eNOS, and 5'-AAT TCC GAT AAC GAA CGA GA-3' and 5'-ATC TAA GGG CAT CAC AGA CC-3' for 18S rRNA. Each PCR reaction was performed with 11.25 µL 2.5× Real Master Mix, 1 µL of both amplification primers, 1 µL cDNA, and 11.75 μL distilled water for a total volume of 25 μL. The PCR protocol was 95 °C for 10 s; 40 cycles of 95 °C for 30 s, 60 °C for 30 s, and 68 °C for 30 s; and 55 °C for 10 s. Duplicate CT values were analyzed with the comparative *C*_t_ (ΔΔ*C*_t_) method. The amount of target (2^−ΔΔ*C*t^) was obtained by normalizing to 18S relative to a calibrator and expressed as the fold difference from the control group [49].

### 4.6. Measurement of NO Levels in Fallopian Tube Cultures

Nitric oxide is produced from l-arginine by NOS, but the half-life of nitric oxide is very short and it is quickly metabolized to nitrate and nitrite. The nitric oxide assay kit (Thermo Fisher Scientific Inc.) used in this study uses nitrate reductase to convert nitrate to nitrite, thus we measure the nitrite concentration to see if the total nitric oxide level released by iNOS and eNOS changes in the culture medium. After 6 and 24 h of *in vitro* incubation, a 50 µL reaction volume with nitrate reductase was used to convert nitrate to nitrite in the 96-well flat-bottomed plate. The nitrate was then detected as a colored azo dye product of the Griess reaction that absorbs visible light at 540 nm and can be measured with a microplate fluorometer (Molecular Devices, Sunnyvale, CA, USA).

### 4.7. Lactate Dehydrogenase (LDH) Release Assay

LDH is a marker for cell apoptosis and is an indicator of the integrity of the cell membrane. If the cell membrane is damaged, LDH will be released. Thus, we measure the LDH level in the culture medium to see if the NO level changes due to cell death. After 6 and 24 h of incubation, the culture medium was collected and analyzed for LDH release using a cytotoxicity detection kit (Roche Applied Science, Mannheim, Germany) following the manufacturer’s instruction. A 50 µL volume was used for reactions with the LDH substrate in the 96-well flat-bottomed plate. Optical density was recorded in a microplate fluorometer at a wavelength of 490 nm. The levels of LDH released into the medium from the cytosol of damaged tissues and cells in each sample were measured in three replicates and the average LDH level was calculated. The percent LDH release per treatment condition was calculated using the formula: (experimental release − background)/(maximum release − background) × 100. The maximum release represents the value from detergent-lysed tubal tissues. The background control represents the colorimetric value of the medium immediately after collecting the tissues.

### 4.8. Statistical Analysis

Results were analyzed with the Statistical Package for the Social Sciences statistical software (SPSS v.19, producer, Chicago, IL, USA) and are expressed as mean ± SEM. The data were compared by one-way analysis of variance (ANOVA). Analysis of qRT-PCR results after the hormonal treatment used a two-way ANOVA to assess the main effects of treatment and time and to identify interactions between them [49]. If significant interactions between the fixed factors were observed, within-group analyses were performed with a one-way ANOVA and the significance of the difference was determined by the Dunnett test. *p* < 0.05 was considered a significant difference.
